# Restricted access to the NHS during the COVID-19 pandemic: Is it time to move away from the rationed clinical response?

**DOI:** 10.1016/j.lanepe.2021.100201

**Published:** 2021-08-18

**Authors:** Daniel K Goyal, Fatma Mansab, Adeeb P Naasan, Amir Iqbal, Colin Millar, Grant Franklin, Stephen Thomas, John McFadden, Derek Burke, Daniel Lasserson

**Affiliations:** 1 Consultant Physician & COVID Co-Lead, Lorn & Islands Hospital, Oban, NHS Highlands, Scotland, UK; Clinical Lecturer, Health Systems, University of Gibraltar, Gibraltar; 2 Researcher, COVID-19 Team, Public Health Gibraltar; Clinical Lecturer, Postgraduate Medical School, University of Gibraltar; 3 Senior House Officer in General Internal Medicine and Covid-19 Response Co-ordinator, Lorn & Islands Hospital, Oban, NHS Highlands, Scotland, UK; 4 Senior Clinical Lead, Covid-19 Remote Monitoring of Patients During Response & Recovery, NHS Grampian, Scotland, UK; 5 Consultant Physician, Covid Co-Lead, Lorn & Islands Hospital, Oban, NHS Highlands, Scotland, UK; 6 Consultant Acute Medicine, Raigmore Hospital, NHS Highlands, Inverness, Scotland, UK; 7 Consultant Respiratory Physician, Raigmore Hospital, NHS Highlands, Inverness, Scotland, UK; 8 General Practitioner, Burnfield Medical Practice, NHS Highlands, Scotland, UK; 9 Head of Clinical Governance and GMC Suitable Person, Gibraltar Health Authority, Gibraltar (formerly Consultant in Paediatric Emergency Medicine and Medical Director Sheffield Children's NHS FT); 10 Professor of Acute Ambulatory Care, University of Warwick; Clinical Lead for Ambulatory Outreach Team, Oxford University Hospital, Oxford, UK

## 1 Introduction

Recently a Lancet Commission examined the future prospects of the NHS in the wake of COVID-19. The report cites poor healthcare capacity and chronic staff shortages as key contributing factors to the UK's inadequate pandemic response. Notable strengths included universal access, the goodwill of staff, and the ability to generate innovative solutions - qualities that are likely to have averted an even deeper national crisis [1].

The prosperity of the NHS is intrinsically connected to the prosperity of the nation. Access to healthcare influences morbidity, mortality, economic activity, and whether or not social restrictions are necessary [2,3]. Public health measures such as timely implementation of social distancing are also important to limit mortality, but going forward it is the capacity to respond in a clinically effective and decisive manner that is vital to diminish the threat associated with the virus [4].

The importance of examining the national clinical response to SARS-CoV-2 cannot be overstated. Arguably the greatest mistake of this pandemic would be failing to prepare for the next. There are also the looming unknowns of SARS-CoV-2 variants [5], the higher rates of Long COVID following more severe disease [6], and the increased healthcare demands associated with delayed presentation of COVID-19 pneumonia [7], [8], [9], [10], [11]. Improving the tolerance of society to background levels of SARS-CoV-2 will require an improved clinical response. With this in mind, we examine one aspect of the UK's clinical response that remains in place today: restricted access to healthcare.

## 2 The rationed response

Part of the poor clinical response to COVID-19 in the UK can be traced back to national policies restricting access to healthcare. Early on in the crisis the national response defaulted to a passive clinical approach [12,13], despite international recommendations to the contrary [14]. UK-wide, patients were advised to stay at home, book a SARS-CoV-2 test, and if concerned consult either an automated online symptom checker or non-clinical telephone triage system [12,13]. Notably, thresholds for onward referral using these new and unproven triage systems were high [15]. Equally concerning, the subsequent automated safety-net advice given to the patient included ‘how to manage breathlessness at home’ [13] – a practice that would have been inconceivable in 2019. This, what became the national COVID-19 clinical pathway, replaced the more typical GP-led community assessment of the infected, breathless patient [16].

As well as clinical contact, oxygen was also rationed [17,18]. At a national level, regardless of local disease prevalence and even for patients without COVID-19, target oxygen levels were reduced. This departure from our usual standards of care was imposed nationwide without any new evidence or revision to our established pneumonia guidelines [17]. This meant patients with severe COVID-19 could be left at home, or sent home, hypoxic and without any treatment or follow-up. [Some localities did identify this gap in the national response and implemented their own follow-up service for high-risk patients who did not meet the new, higher thresholds for admission [19].

The rationale for such a passive, restricted national clinical response to the disease is not entirely clear. It may have been a preemptive rationing of healthcare; an attempt to concentrate limited clinical resources to those in most clinical need. But acute medical problems generally follow a different logic. Any offsetting of the healthcare burden achieved by restricting access to clinical contact early on in the disease is lost when patients - albeit fewer in number - present more severely unwell further on in the course of the disease [7], [8], [9]. A ten minute clinical assessment can quite easily become many hours of clinical time if the opportunity to intervene early is missed. And, a short, uncomplicated hospital stay can quite easily become a complicated and protracted one, if treatment is delayed.

The more typical early intervention approach normally applied to pneumonic illnesses is both clinically effective and resource-savvy [7]. It starts with a basic medical principle: pneumonia responds poorly to a lack of attention [7], [8], [9], [10], [11]. Patients who present late have greater healthcare needs and require longer inpatient stays [8,9]. Pneumonia detected early is simpler to treat, with less need for high dependency care, shorter hospital admissions, and less morbidity and mortality [8], [9], [10], [11]. Most patients do not develop COVID-19 pneumonia, but older adults and some vulnerable patients carry a substantive risk of developing pneumonia and a high mortality[20]. And all adults carry an increased risk of higher post-pneumonia complications (e.g., post-COVID syndromes) when treatment is delayed and the opportunity to prevent disease progression is missed [6]. Failure to respect these tenets of clinical care and capitalise on the opportunity to intervene early will serve only to deepen the extent and duration of the healthcare crisis.

International guidelines do not support the UK's passive clinical approach to SARS-CoV-2. The WHO issued guidelines in March 2020 recommending a clinical assessment be offered to all patients with suspected or confirmed COVID-19 [14]. The UK has yet to meet those standards. The WHO also produced similar guidelines directed at ‘resource-restricted’ countries [21]. All four UK nations have failed to meet those standards too [22], [23], [24], [25]. Even now, in the UK, patients with COVID-19 – suspected or confirmed – are still not offered an initial clinical assessment or follow-up, be it remote or otherwise. The public can still order a diagnostic test without clinical supervision, are still triaged through an automated or non-clinical pathway, and astonishingly, are still offered advice (and now a video) on how to manage breathlessness at home without ever having seen or spoken to a doctor or nurse [12,13,[22], [23], [24], [25]]. Of critical concern, at the time of writing, older adults and vulnerable patients are held to the same pathway [22], [23], [24], [25], despite our awareness of ‘silent hypoxia’ and the mortality rate of the vulnerable and older COVID-19 patients [20]. These standards are substantially below those expected in the UK and internationally, and need to be addressed.

## 3 Basic healthcare provision

Early in 2020, the WHO issued pandemic-specific technical guidelines recommending an expansion to basic healthcare provision [26]. An expanded basic healthcare capacity typically refers to an increase in inpatient bed capacity with the accompanying increase in staffing; although, these basic secondary care services can sometimes be provided in the community via outreach services [27]. Basic healthcare facilities enable at-risk or deteriorating patients access to clinical monitoring and the mainstay of treatment for COVID-19 pneumonia. Dedicated basic care facilities also provide effective isolation for the most infective cases and helps alleviate the fear of nosocomial spread [26].

UK policy focused instead on expanding intensive care unit (ICU) capacity. Basic healthcare capacity was fashioned by curtailing other essential healthcare services. The existing NHS bed quota were used to provide beds for COVID-19 patients and no actual increase in basic healthcare capacity has, to this day, occurred. Indeed, together with the effects of social distancing for infection control purposes, the NHS suffered an 8% reduction in usable inpatient bed capacity during the pandemic [28].

The rationale behind the UK national policy to expand only HDU/ICU care and not expand basic healthcare provision is not entirely clear. Pneumonia is effectively managed by preventing disease progression. Intervening early, supplemental oxygen, thrombosis prophylaxis, and instigating treatments as soon as the patient is eligible – all of which can be done in a basic healthcare setting – [10,11], limits progression of disease, improves recovery times, and conserves the high-intensity clinical areas for the small proportion of patients who do not respond to these basic interventions [7], [8], [9]. Waiting for patients to deteriorate and then providing care is neither effective nor efficient.

## 4 An evolving clinical response

Chronic underfunding is likely at the heart of the restricted and rationed clinical pandemic response [1]. Asking an overstretched and historically neglected primary care service [29] to provide clinical contact to all patients with SARS-CoV-2 is challenging without appropriate levels of funding and a reprioritisation of the role of the GP. Already, remote consults [30], greater use of non-medical clinical staff [31], and automated remote monitoring services [32] have been implemented in some parts of the country to try and offset the additional demands on primary care, however access to these services is limited [33].

Innovative ‘COVID Hubs’ have been developed in some parts of the country to enable segregated and dedicated clinical support to COVID-19 patients [34]. Unfortunately, while reducing the risk of viral transmission and providing a route for clinical contact, COVID Hubs do not seem to have moved beyond the passive clinical posture underpinning the UK national clinical response. Patients typically have no direct access to the Hubs - even COVID-19 positive patients -, and Hubs typically do not provide any proactive outreach to high-risk positive patients [35]. The system remains reactionary. The national default continues to be for the public to self-isolate and, if concerned, to utilise the online and telephone triage of NHS 111 [36].

A number of localities developed virtual wards in an attempt to provide some follow-up for high-risk patients who had presented to the emergency department or GP [19]. In Southampton University Hospital, for example, both GPs and secondary care physicians managed at risk COVID-19 positive (or suspected) cases in the community, often remotely. This appears to have been relatively successful in providing improved follow-up for patients who had already gained access to healthcare (although controlled data is currently lacking) [37]. Such local success with virtual wards has led to national recommendations - at least in England [38] - to develop virtual wards as part of the secondary care of the COVID-19 patient. There is though, no contractural arrangement for healthcare trusts to provide virtual wards and as such no specific funding [39]. Together with the passive national clinical response to COVID-19, the impact of virtual wards on improving access to healthcare and improving the resilience of the NHS is likely to remain limited.

Remote monitoring has also been developed in some parts of the UK [22,23]. This includes using an automated system for monitoring symptoms and, in higher risk patients, pulse oximeters at home for monitoring oxygen levels [32]. It is a welcomed, albeit not yet evidence-based, intervention, likely providing an improved level of community vigilance for those with COVID-19. Importantly, enrolment in such monitoring services does not occur at the point of diagnosis, not even for the vulnerable or older patient. To access such services patients must still identify their own deterioration and meet the online or telephone triage criteria for onward referral [22,23]. The costs for monitoring the data generated and hence the patient has also not yet been adequately met, the burden falling mainly on primary care [39]. Again, within the passive model of care currently in place and subsequent passive enrollment of patients onto home monitoring services, and without a contractural obligation to provide the service, remote monitoring is unlikely to achieve the cost savings and healthcare resilience benefits it could.

## 5 Pandemic responsiveness

While a degree of restricted access to healthcare is expected during a pandemic of this magnitude, the extent of healthcare restrictions that occurred in the UK, and the fact they are continuing, is concerning. Case numbers of severe infections are lower, treatment options have improved [10,11], and yet patients with COVID-19 are still not offered clinical follow-up, not even older or vulnerable patients. This suggests the national clinical response lacks coordination and adaptability – there is an inadequate pandemic responsiveness.

Triaging is a clinical activity where clinical judgement is deployed to determine the most appropriate next step in a patient's journey, often under resource constraints. The skill in clinical triage is in identifying those patients who will benefit from the limited available resources. Clinical triage is not static. It changes, often daily, often in response to evolving knowledge and experience, and certainly in response to local resource availability. Effective triage benefits both patient and the healthcare service, as resources are directed where they are likely to work, and, crucially, at the moment at which they are most effective [40].

During this pandemic, the clinical aspect of triaging has been substituted by a ubiquitous national strategy of using the NHS 111 online and non-clinical telephone triage service, replacing clinical judgement with predetermined thresholds for onward referral. Unless the thresholds for onward assessment are set low, many opportunities to prevent disease progression will be missed, and with it the opportunity to reduce mortality, prevent post-pneumonia complications, and prevent prolonged and protracted hospital admissions. In the UK, the thresholds for recommending any clinical contact are set high, and not just for the areas suffering a surge of infections, but for the entire nation - restricting access to care even where healthcare demands are relatively low [15].

Ideally, and as recommended by the WHO [14,21], all patients with suspected or confirmed SARS-CoV-2 should undergo clinical triage and follow-up. With the pressures on the NHS and variation in SARS-CoV-2 case numbers this will not always be possible. Some adaptability will be needed. In terms of mortality savings and optimising the flow of patients through inpatient care - protecting ICUs and the NHS - adaption should occur in relation to risk, not by automated systems or non-clinical staff trying to determine disease severity.

Singapore has at times adapted their response based on risk. Typically, in Singapore, any member of the public can book a same day appointment at one of 900 public health clinics (run by primary care), receive a clinical assessment, a SARS-CoV-2 swab, and be triaged home with follow-up, or referred to a dedicated secondary care service for further assessment [41,42]. During a surge of SARS-CoV-2 cases in April 2020, Singapore's online triage tool was changed to advise the non-breathless, young patient to stay at home and to contact their GP if symptoms hadn't improved after three days [15]. While still a compromised response - and one that was quickly reversed when case numbers fell -, it permitted some low-risk relief to the healthcare service.

A dynamic and adaptable pandemic response is vital if we are to maximise the clinical resources available. A wholesale approach to triage is neither evidence-based nor is it likely to succeed [40]. Access to healthcare saves lives [2,3], and timely access to healthcare saves lives and resources [7], [8], [9], [10], [11]. Directing patients to the most appropriate level of care can only be achieved if the local options for care are considered. Compromises may need to be made, but we also must accept they are compromises, and wherever possible re-address the balance back to our more typical, dynamic clinical approach.

## 6 Time to move away from the rationed response

A number of European nations have maintained direct clinical contact for patients with confirmed or suspected COVID-19. Switzerland has maintained direct access to GPs, who undertake the majority of COVID-19 patient triage [43]. Germany has also directed patients to primary care, where a reported 85% of COVID-19 cases have been managed. Germany has also delivered direct clinical access through community ‘Corona Clinics’; delivered outreach services to reach the more vulnerable patients via ‘Corona Taxi's’, and expanded basic healthcare capacity to offload the pressures from both primary and secondary care facilities [44].

The current UK national clinical pathway for patients with suspected COVID-19 has improved but remains passive and reactionary, and overly reliant on existing, overstretched services. This bodes poorly for how the UK will manage any ensuing wave of SARS-CoV-2 variants or future pandemics. Basic healthcare capacity (inpatient bed capacity) remains lower than before the pandemic [45]. Clinical triage does not occur at the point of suspected COVID-19, nor does it even occur when COVID-19 is confirmed. Self-isolation and then contacting NHS 111 if symptoms become concerning remains the default advice to the public [36]. Remote monitoring has been recommended in England and some parts of Scotland, but only those patients who identify their own deterioration currently have a chance of enrolment and there is no contractural obligation for its provision [22,23].

An improved UK response should more closely follow the WHO technical guidelines for triage and management of COVID-19 [14,21]. Invariably this will include more dedicated primary care time to contact at least the at-risk patient groups at the point of diagnosis; a dedicated clinical follow-up service; a home outreach service for the most vulnerable of patients, and crucially, the capacity to admit the deteriorating or high-risk patient. These pathways would need to be clear to both patient and clinician, and will require additional funding and political support to achieve universal access.

In the absence of such support, we expect the lower numbers of older and vulnerable patients who contract SARS-CoV-2 could (and should) still be prioritised into existing primary care pathways for direct - be it remote or otherwise - clinical contact at the point of diagnosis.

## 7 Conclusion

COVID-19 has highlighted an NHS with insufficient resources and not enough capacity or healthcare resilience to keep society running during a pandemic. The pressures on NHS capacity prior to the pandemic and the inadequate expansion in healthcare provision since, likely mean the UK will struggle to achieve international standards (even those of a low-resource setting), at least not in a reasonable timeframe. Without the essential access to healthcare we lose the opportunity to prevent disease progression and with it the opportunities to save lives, prevent disability, and to more effectively safeguard the NHS.

Necessity has spawned potentially useful innovations. Dedicated COVID assessment centres, remote assessments, virtual wards, and remote monitoring are likely to achieve clinical care closer to pre-pandemic standards of care while saving hospital admissions and clinical time. However, the lack of real-world funding and therefore contractural obligation to deliver such services, together with the most crucial of healthcare gaps - the lack of clinical triage, clinical follow-up, and a safe level of inpatient capacity - severely curtail the benefit these innovations can achieve.

Given the challenges of delivering coordinated pandemic responsiveness at the national level, it may be neither wise nor – in the acute setting – necessary to wait for an adequate national response to these healthcare needs. It may be more prudent for localities - COVID-19 service providers, GP practices, local healthcare authorities - to define their own healthcare prioritisation. If so doing, we urge a more proactive clinical posture and, at least, the minimum standard of an initial clinical assessment and clinical follow-up to all older and vulnerable patients who contract SARS-CoV-2.

## 8 Contribution statement

All authors contributed to the discussion and conception of the viewpoint. AI, DL, & JM provided review and expertise for primary care COVID-19 pathways; DL also provided review and expertise for virtual wards and ambulatory pathways; DG, CM & GF provided review and expertise for acute and general medicine pathways and follow-up services; FM for public health and pandemic responsiveness; AN for inpatient pathways; ST for respiratory medicine, and DB for emergency medicine and clinical governance. DG and FM wrote the initial draft and first review with input from DL. All authors provided input on the final draft before submission.

## Declarations of competing interest

We have read and understood the Lancet Regional Health - Europe policy on declaration of interests and have no relevant interests to declare.
